# Bibliometric analysis of renal cell carcinoma with venous tumor thrombus

**DOI:** 10.7150/ijms.98359

**Published:** 2024-08-12

**Authors:** Jinbin Xu, Weijen Lee, Shoudong Yang, Shuntian Gao, Yuedian Ye, Gengguo Deng, Weixing Zhang, Jinming Di

**Affiliations:** 1 Department of Urology, The Third Affiliated Hospital, Sun Yat-sen University, Guangzhou, Guangdong, China.; 2 Department of Urology, Chashan Hospital of Dongguan, Dongguan, Guangdong, China.

**Keywords:** Renal cell carcinoma, Venous tumor thrombus, Thrombectomy, Neoadjuvant immunotherapy, Bibliometric analysis

## Abstract

**Objectives:** To identify the cooperation of authors, countries, institutions and explore the hot spots regarding research of renal cell carcinoma with venous tumor thrombus.

**Methods:** Relevant articles were obtained from the Web of Science Core database (WoSC) from 1999 to 2024. CiteSpace was used to perform the analysis and visualization of scientific productivity and emerging trends. Network maps were generated to evaluate the collaborations between different authors, countries, institutions, and keywords.

**Results:** A total of 2180 related articles were identified. We observed an increased enthusiasm in related fields during the past two decades. The USA dominated the field in all countries, and the University of Miami was the core institution. Ciancio G might have a significant influence with more publications and co-citations. Current research hotspots in this field mainly included thrombectomy, tyrosine kinase inhibitors, immune checkpoint inhibitors, vena cava inferior, and microvascular invasion. Thrombectomy complications, thrombectomy survival outcome, and preoperative neoadjuvant immunotherapy represented the frontiers of research in this field, undergoing an explosive phase.

**Conclusion:** This is the first bibliometric study that comprehensively visualize the research trends and status of RCC with VTT. We hope that this work will provide new ideas for advancing the scientific research and clinical application.

## Introduction

Renal cell carcinoma (RCC) is the primary malignant tumor of the kidney, representing 2-3% of all new cancer cases annually, with clear cell renal cell carcinoma (ccRCC) making up nearly 90% of all diagnosed subtypes 1. Recurrence and metastasis were observed in a part of RCC cases after surgery 2. Tumor extension into the venous circulation, as one kind of metastasizes, is a well-described phenomenon of RCC, occurring in 4% to 10% of cases 3. There are numerous potential factors associated with vascular invasion, including a large tumor diameter, advanced clinical tumor stage, malignant tumors pathological features. And tumors with lymphatic invasion, tumor microenvironments are also crucial. The vascular metastasis of renal cell carcinoma primarily involves the renal vein, the central adrenal vein, and the small blood vessels around the tumor, resulting in venous filling defects in imaging and the formation of tumor thrombus in tissue. The level of tumor thrombus is defined by imaging characteristics based on the Mayo Clinic classification as described by Neves and Zincke 4.

Patients with tumor thrombosis have worse overall survival and disease-free survival, then radical resection dominates the treatment option. Since the first case series was reported by Skinner et al in 1972 5, radical nephrectomy (RN) with venous tumor thrombus (VTT) thrombectomy has been the only standard treatment option for RCC with inferior vena cava (IVC) tumor thrombus, yet the intricate nature of the procedure and the significant morbidity limited its widespread adoption 6, 7. Hence, from conventional open RN to laparoscopic procedure, and then, since first reported a decade ago, robotic-assisted radical nephrectomy with inferior vena cava thrombectomy (R-CT) with a more minimally invasive feature and less incidence of postoperative complication, has become the alternative option for this disease. In a recently published meta-analysis, Garg H systematically reviewed potential articles and identified 28 articles describing R-CT and open radical nephrectomy with inferior vena cava thrombectomy (O-CT). The analysis indicated that R-CT was linked to a lower blood transfusion rate (18.4% vs. 64.3%, *P* < 0.002) and a lower complication rate (14.5% vs. 36.7%, *P* < 0.005) 8. Underlying the mechanisms driving tumor thrombus formation to design the effective neoadjuvant therapy on target for downstaging tumor thrombus or decreasing the perioperative morbidity in thrombectomy is another direction. Due to the superior efficacy of sunitinib in other solid tumors, Tyrosine kinase inhibitors (TKIs) have gradually entered the center of tumor immunotherapy for RCC since 2004 9. In recent years, existing research has gone further to explore the mechanism of thrombosis at the cellular level. Shi Y decoding the multicellular ecosystem of VTT at single-cell RNA sequencing 10, the outcome showed a higher level of progenitor exhausted phenotype tissue resident CD8+ T cells in tumor thrombus. Additionally, they also found that macrophages, malignant cells, endothelial cells, and myofibroblasts from IVC enhanced the remodeling of extracellular matrix. Niu S used whole-exome sequencing to analyze VTT tissues and relevant matched primary tumor tissues 11. He found that BAP1, KDM5C, CELSR2, and TET2 alterations were higher presented in VTT tracks.

CiteSpace is a web-based Java application for bibliometrics analysis and visualization 12. It uses quantitative and statistical methods to analyze the production and dissemination of research literature, involving collecting, organizing, and analyzing bibliographic data, like multi-dimension analysis (countries/regions, institutions, authors), cluster analysis and keywords citation burst analysis. It can identify and quantify the impact of research and can support the identification of research trends, emerging fields, and collaborations 13. The technology of RCC with VTT has made great progress, while there is still a lack of visualization literature. This study investigates the research RCC with VTT development based on CiteSpace document visualization analysis.

## Methods

### Data source and search strategy

The Web of Science core database was retrieved by the following search string: TS = (("carcinoma, renal cell" OR "renal clear cell carcinoma" OR "renal cell cancer" OR "adenocarcinoma of kidney" OR "clear cell renal carcinoma") AND ("tumor thrombus " OR " vascular invasion " OR "vascular involvement" OR " microvascular invasion ")). Articles that met the following criteria were enrolled: (1) the publication span is between 1999 to 2024; (2) To facilitate further analysis of literature content, only regular articles written in English were included.

### Statistical analysis

CiteSpace 6.1.R3 was utilized for conducting bibliometric analysis to generate visual knowledge maps encompassing countries, authors, institutions, and keywords. These visualized maps comprise nodes and links, where the links between nodes denote cooperative/co-occurrence or joint relationships. Nodes are scaled based on publication volume, with larger nodes representing higher publication counts. The color and thickness of the node's circle signify varying publication numbers across different periods. The software was configured with specific parameters: time slicing from January 1999 to March 2024, with each year serving as a time slice. The G-index was employed as the analytical metric. Pruning methods included the merged network, pathfinder, and pruning sliced networks were applied. Modularity Q (Q) and Weighted Mean Silhouette (S) were used to assess the degree of clustering, where Q > 0.3 indicates a significant clustering structure, and S > 0.5 suggests acceptable clustering results. Authors, institutions, and keywords were treated as distinct node types for conducting a visual atlas analysis within the included literature.

## Results

### Analysis of Publication literature output

**Figure 1:**
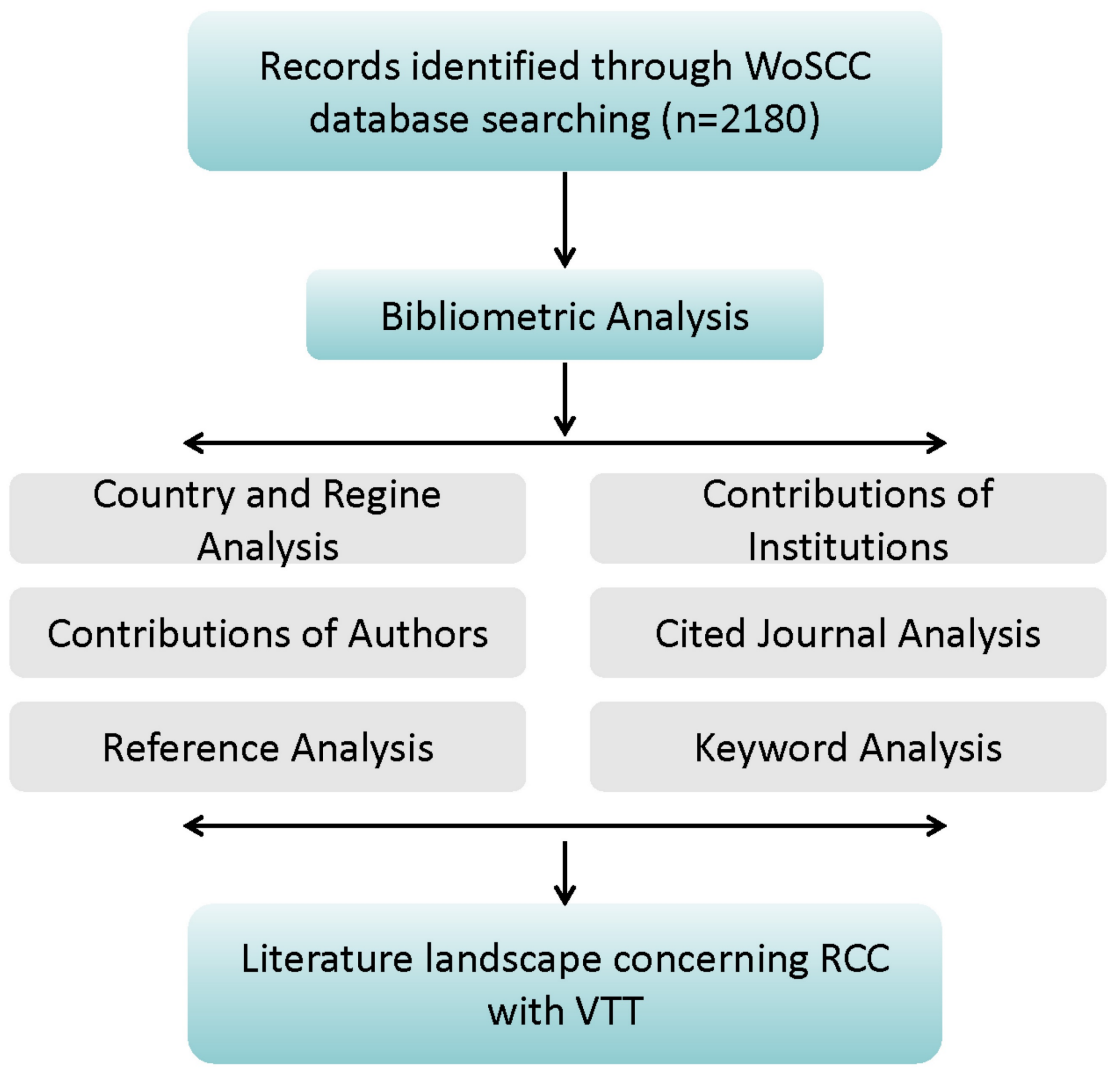
Flowchart of literature selection and scientific bibliometric analysis.

**Figure 2:**
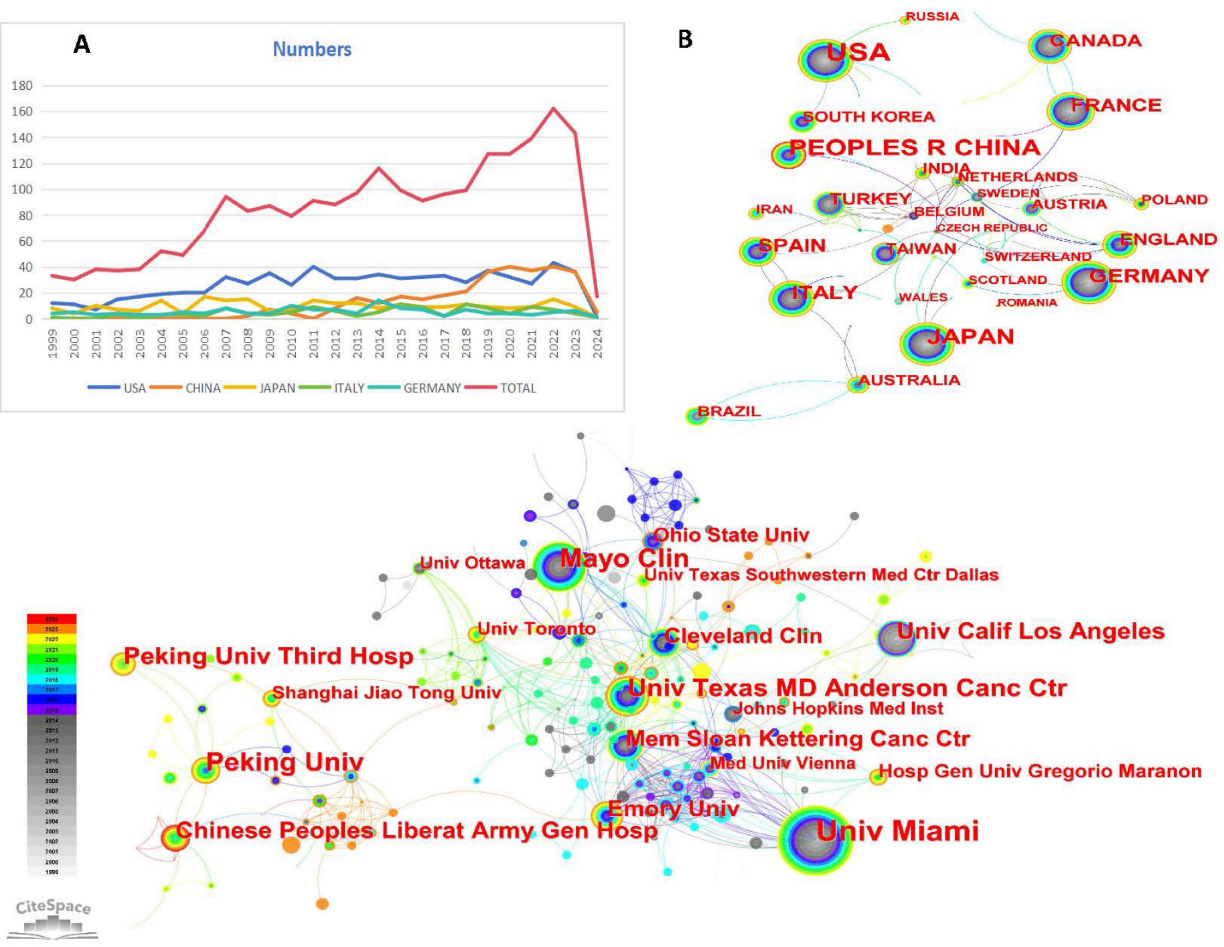
(A) The output of articles about RCC with VTT in top five countries and global publication from 1999 to 2024, (B) the countries/regions collaboration network of RCC with VTT, (C) the institutions collaboration network of RCC with VTT.

As illustrated in **Figure 1**, this study encompassed 2180 conventional articles. **Figure 2A** presented the yearly publication counts concerning RCC with VTT. The number of publications rose from 33 in 1999 to 143 in 2023, indicating a consistent growth in research interest. In 2021, 127 papers were published. The peak in the number of publications (n=162) occurred in 2022, representing 7.43% of the total publications. Citations have been on a rapid and continual rise each year.

### Analysis of countries/regions and institutions

In the landscape of research on RCC with VTT, the collaborative efforts of 73 countries/regions and 546 institutions have significantly enriched the body of related articles. The intricate networks formed by these countries/regions and institutions were represented in **Figure 2B** and **Figure 2C**, shedding light on the global connectivity and knowledge exchange in this field. The dominance of the top 10 countries and institutions contributed 1851 articles (84.91%) and 388 articles (17.80%), respectively, underscoring their significant impact on the scholarly discourse. Examining the countries/regions network with its 73 nodes and 91 links (Density=0.0346), we observed a web of connections indicating collaboration and information flow. The inter linkages among countries, cooperative structure appeared to retain a degree of looseness, suggesting opportunities for further strengthening partnerships and knowledge sharing. The USA emerged as a front runner both in terms of publication volume and centrality (centrality=1.06), with 676 articles leading the pack. Following closely were China (315 papers), Japan (248 papers), Germany (136 papers), and Italy (120 papers).

The collaboration network among institutions in the study of RCC with VTT had 546 nodes and 871 links, the network showcased a robust exchange of knowledge and expertise, as evidenced by its density of 0.0059 (**Fig. 2C**). Among the myriad institutions contributing to this research domain, the top 5 institutions stood out for their significant contributions. Leading the pack was the University of Miami, with an impressive 82 papers, followed by the Mayo Clinic (49 papers), Peking University (46 papers), the University of Texas MD Anderson Cancer Center (41 papers), and Peking University Third Hospital (35 papers). These institutions have emerged as key players in advancing the understanding and treatment of RCC with VTT through their prolific research output. Centrality in a network context measures the importance or influence of a node based on its connections to other nodes. The first four mentioned institutions exhibited greater centrality values, surpassing the threshold of 0.01. Their focused research efforts and extensive collaborations have positioned them as key hubs in the dissemination and advancement of knowledge in this complex medical domain. Further details can be found in** Table 1**.

### Analysis of cited journals

**Figure 3:**
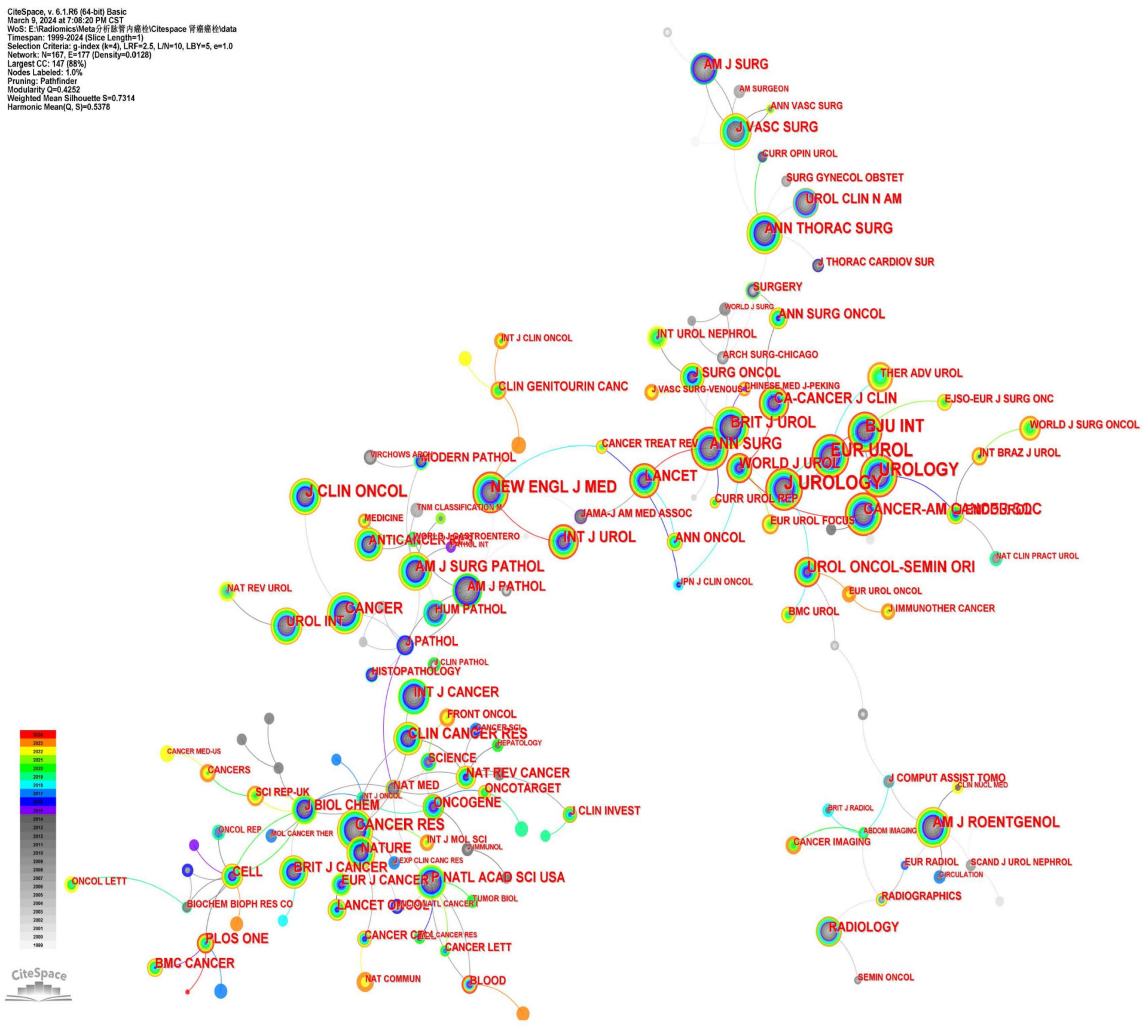
The cited Journals collaboration network.

The study analyzed 2180 articles from 167 journals (**Fig. 3**). **Supplementary Table 1** presented the top 10 journals based on publication quantity along with their latest 2023 impact factors (IF). The most cited journal was J UROLOGY (1330 citations), followed by EUR UROL (1016 citations), UROLOGY (938 citations), and BJU INT (868 citations). Seven out of the top ten journals were in the first quartile (Q1) of the Journal Citation Reports (JCR). Among these journals, five were published by US-based publishers, while the remaining two were from the UK and the Netherlands, respectively.

### Authors and co-cited authors

**Figure 4:**
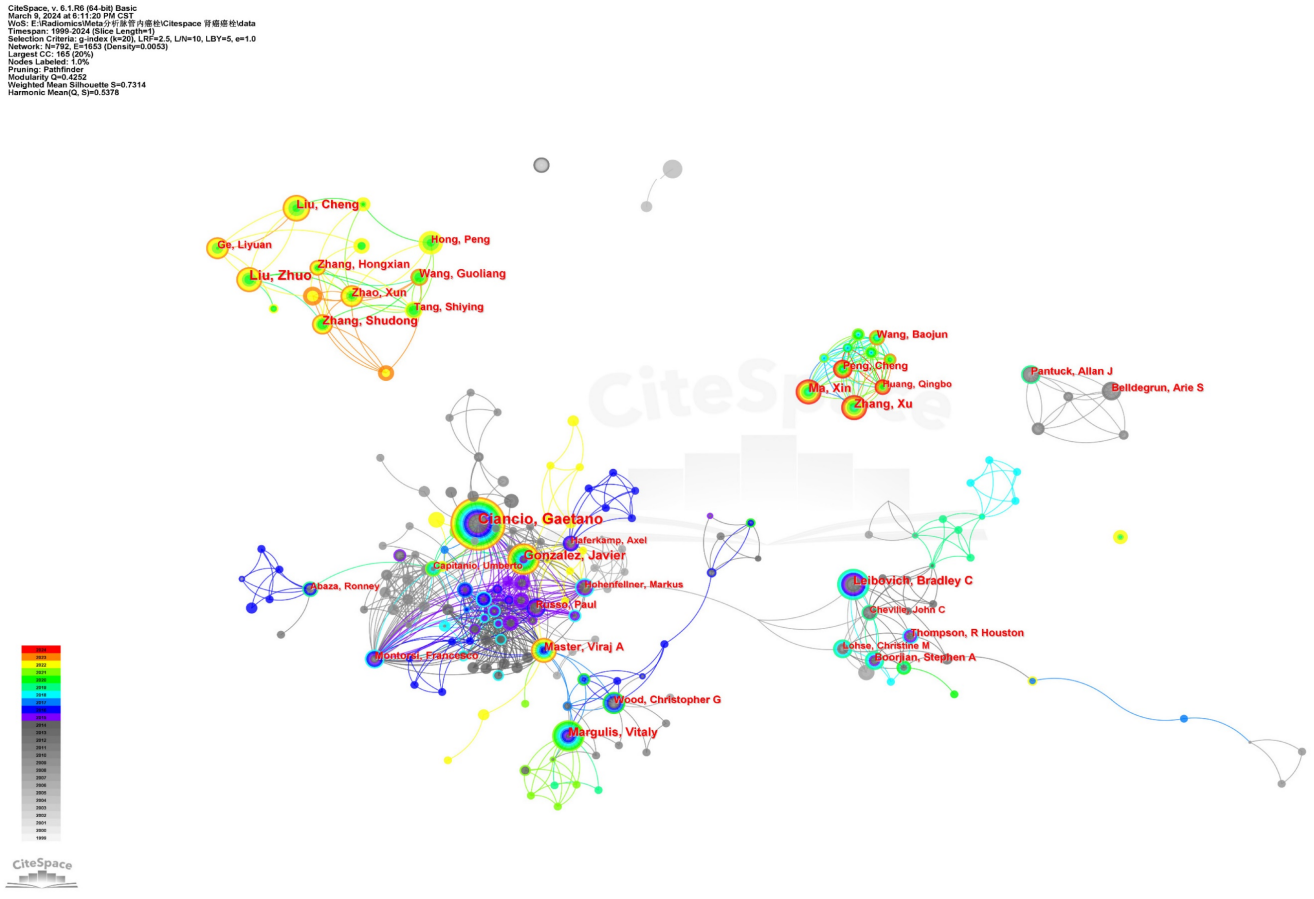
The author collaboration network of RCC with VTT.

**Figure 5:**
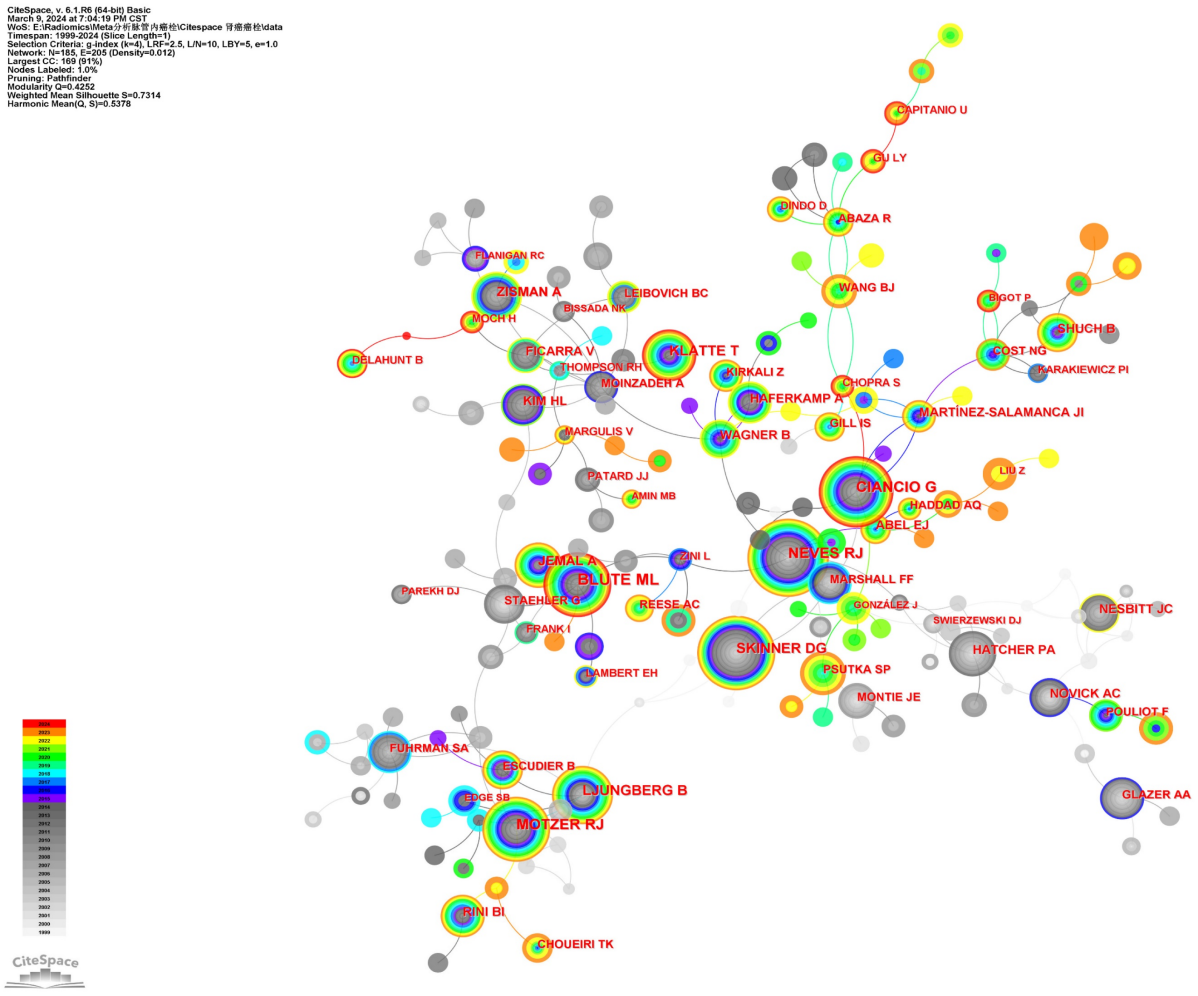
The cited author collaboration network of RCC with VTT.

A total of 792 core authors with 573 links were observed in **Figure 4** (Density=0.0053). The top five authors with the most publications were Ciancio G (72 papers), Ma L (61 papers), Liu Z (43 papers), Zhang S (40 papers), and Gonzalez J (35 papers). In **Figure 4**, each node represented an author, with the size of the circle indicating the number of articles published by that author. The lines connecting the circles depict the co-occurrence relationships between authors. From the author network, we could get the message that Ma X, Zhang X, Peng C, Wang B, and Huang Q closely cooperated from 2019 to now. Ciancio G worked closely with Gonzalez J, Haferkamp A, Capitanio U, Hohenfellner M, Master VA, and Montorsi F. Zhang S, Zhao X, Liu Z, Wang G and Tang S closely cooperated in recent years. Leibovich BC worked closely with Cheville JC, Thompson RH, Lohse, CM, and Boorjian SA. The co-cited authors' visualization map with 185 nods and 205 links in it was given in **Figure 5** (Destiny = 0.012). The top 5 co-cited authors were Blute ML (360 citations), Ciancio G (307 citations), Neves RJ (263 citations), Motzer RJ (257 citations), and Skinner DG (229 citations). The detailed results could be accessed in **Table 2**.

### Co-cited references and references bursts

**Figure 6:**
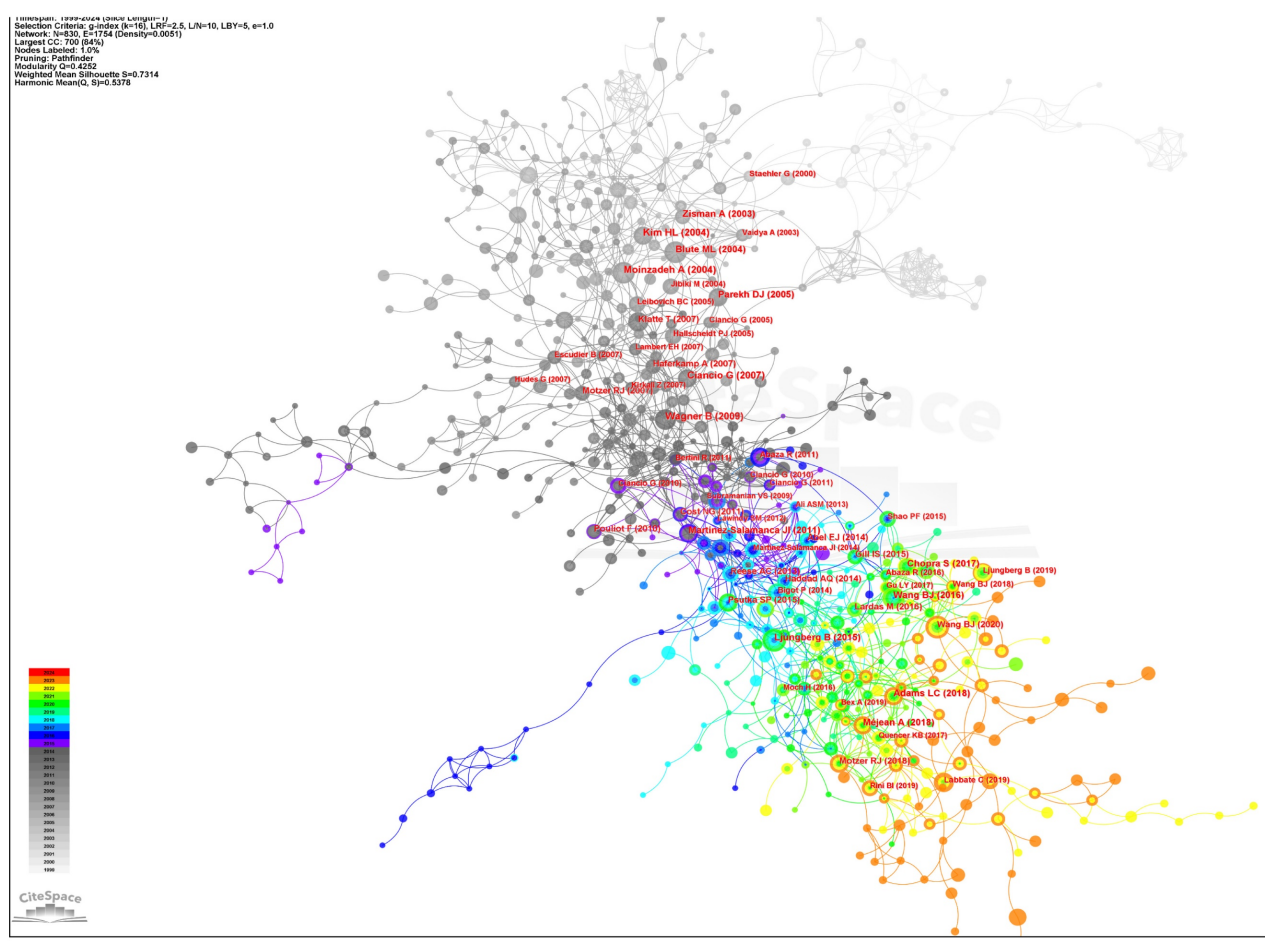
The cited reference collaboration network of RCC with VTT.

**Figure 7:**
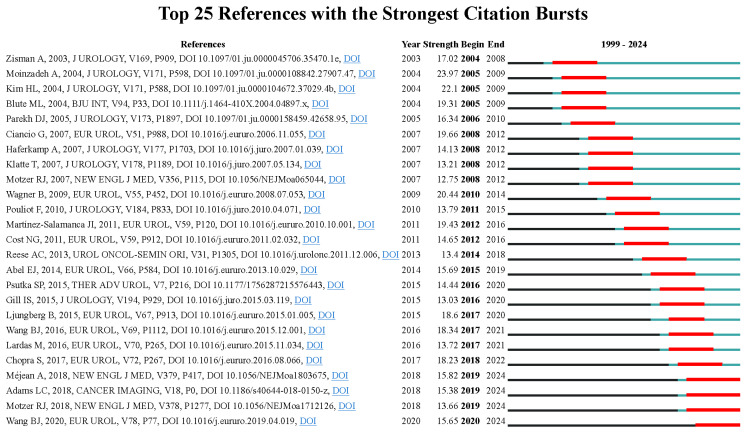
The top 25 references with the strongest citation bursts in the co-citation network.

Co-citation analysis indicates that two references appeared in the reference list of a third citation article, establishing a co-citation relationship. The corporation network map which had 830 nods and 1754 links was shown in **Figure 6** (Destiny=0.0051). Most articles were cited 0~10 times. The top ten co-cited references were demonstrated in detail in **Table 3** 14-23. The article “The level of thrombus in the IVC and survival time” was published in 2004 with 52 citations 14, followed by the article “Prognostic value of renal vein and IVC involvement in RCC” with 49 citations 15, article “Prognostic significance of the level of venous involvement in RCC ” with 48 citations 16, article “Surgical management of RCC with VTT in the Renal” with 43 citations 24, and article “Robot-assisted level II-III IVC tumor thrombectomy ” with 42 citations were the 5 highest citation papers 18. These studies primarily examined the correlation between VTT extension levels and survival duration among patients with RCC. The findings indicated that tumor thrombus level is an independent predictor of survival. None of them had greater centrality (> 0.1), suggesting their research focus was not concentrated. In CiteSpace, nodes with more than 0.1 mediation centrality become the key points 25. **Figure 7** showed the top 25 references with the strongest citation bursts. The last four papers, focus on TKIs and immune checkpoint inhibitors (ICIs) in metastatic RCC 26, 27, MRI imaging for predicting IVC invasion in RCC 28, and robot-assisted IVC thrombectomy 18, has seen a surge increase in citations since 2019, reflecting some of the current research focal points.

### Analysis of co-occurring keywords and cluster

**Figure 8:**
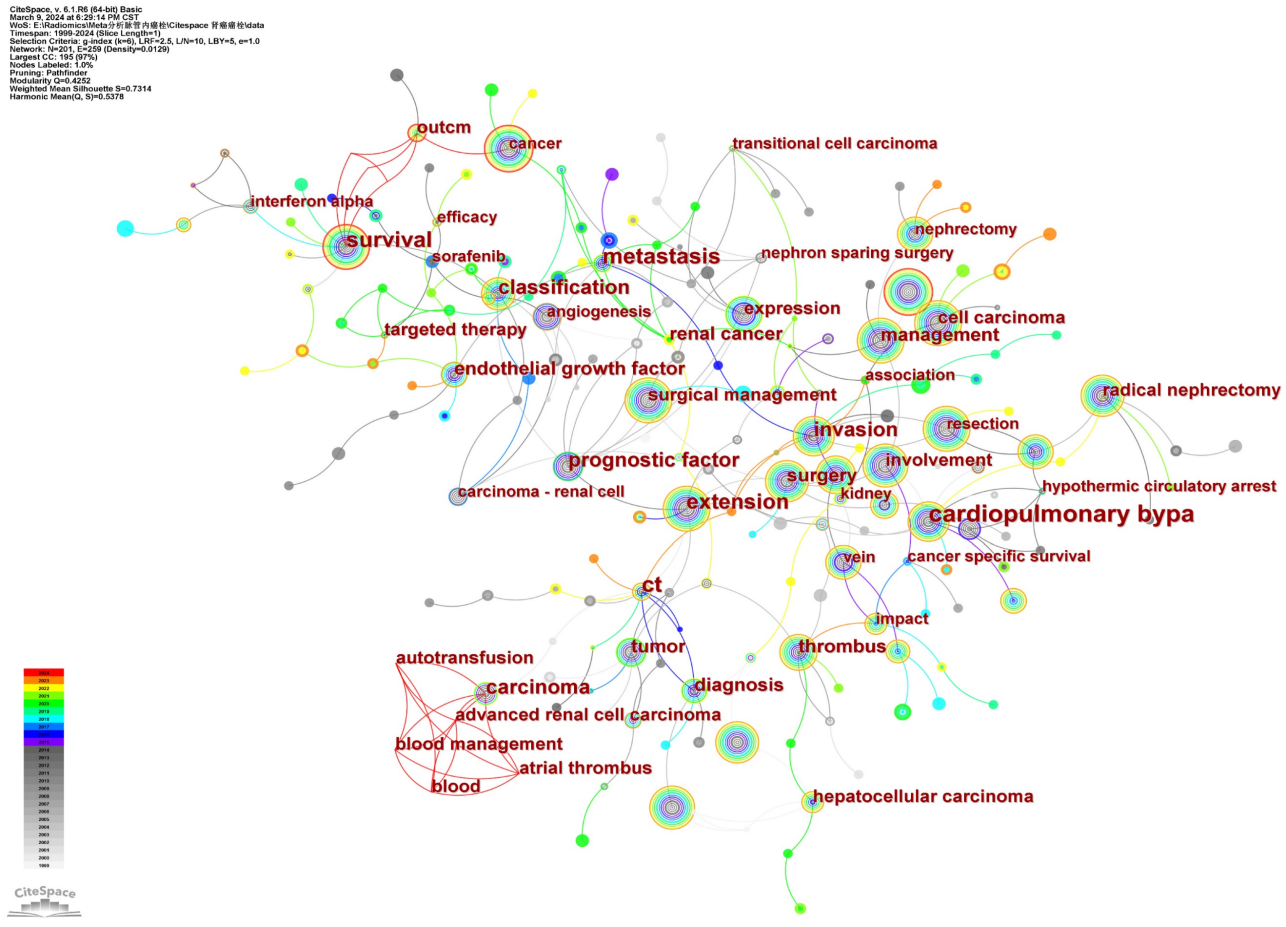
The keyword collaboration network of RCC with VTT.

**Figure 9:**
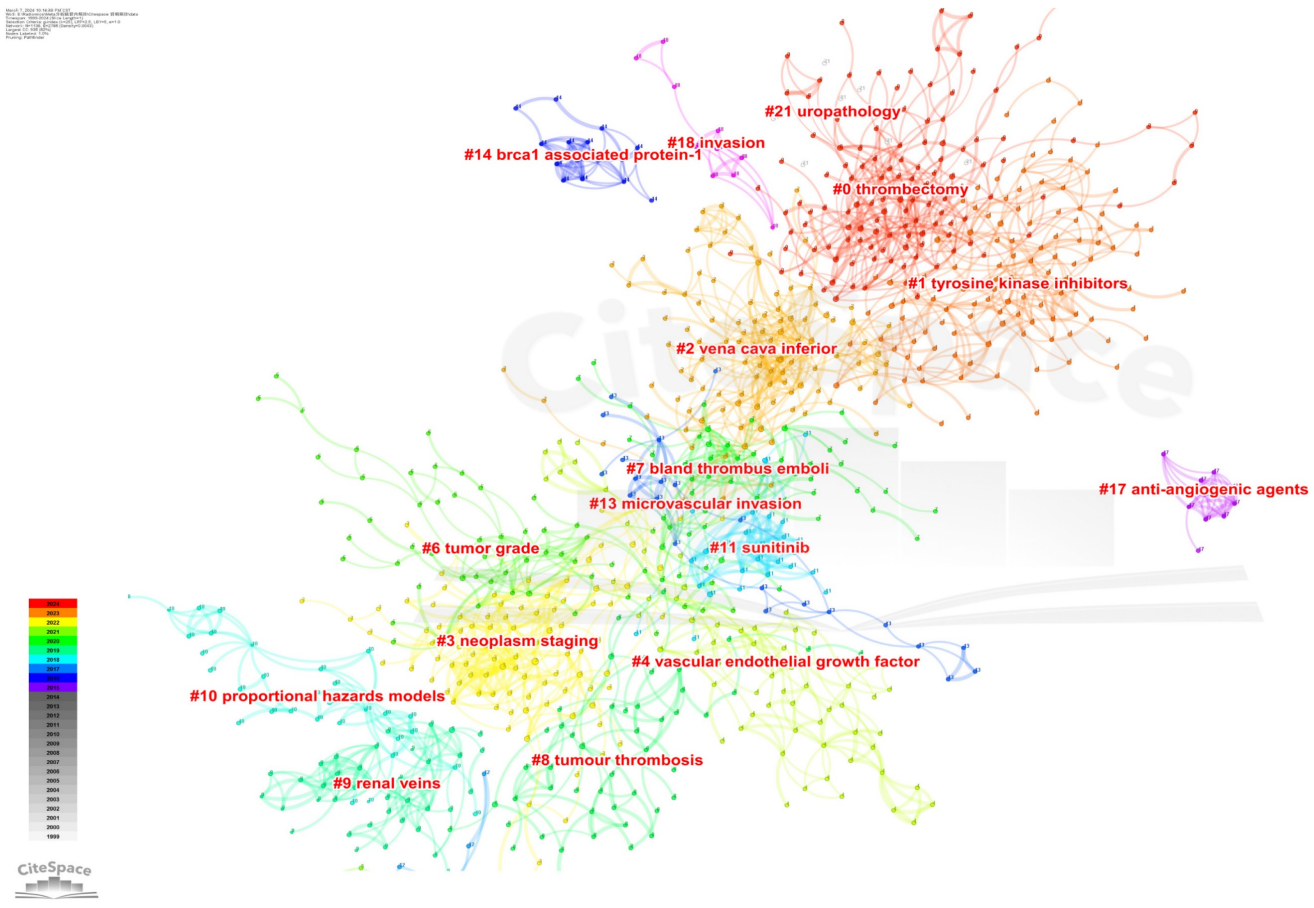
The cluster map of keywords on RCC with VTT.

High-impact keywords signify the authoritative status of related research content within the field, while high-frequency keywords indicate popular topics in research. By employing keyword co-occurrence analysis and burst detection, we can more effectively discern the evolving trends in research topics over time and capture emerging research hotspots. We visually analyzed the keywords in the included literature. There were 201 core keywords with 259 links on the map (**Figure 8**). Keywords with higher frequency in this study included renal cell carcinoma (896), cancer (473), tumor thrombus (349), inferior vena cava (316), radical nephrectomy (288), survival (284), and surgical management (266). More details were shown in **Table 3**. Among these keywords, renal cell carcinoma, cancer, survival, and surgical management had a centrality greater than 0.1, suggesting that the research content on these topics were more focused. More closely, we made these keywords into relevant clusters, and the weighted mean silhouette of each cluster was above 0.5, manifesting a credible result, the network map was shown in **Figure 9.** Thrombectomy, tyrosine kinase inhibitors, vena cava inferior, neoplasm staging, vascular endothelial growth factor, tumor grade, and bland thrombus emboli, have emerged as the central areas of research interest in this field in the most recent studies. These key clusters have garnered significant attention and are driving advancements in understanding and treating conditions related to vascular health and cancer progression.

**Figure 10:**
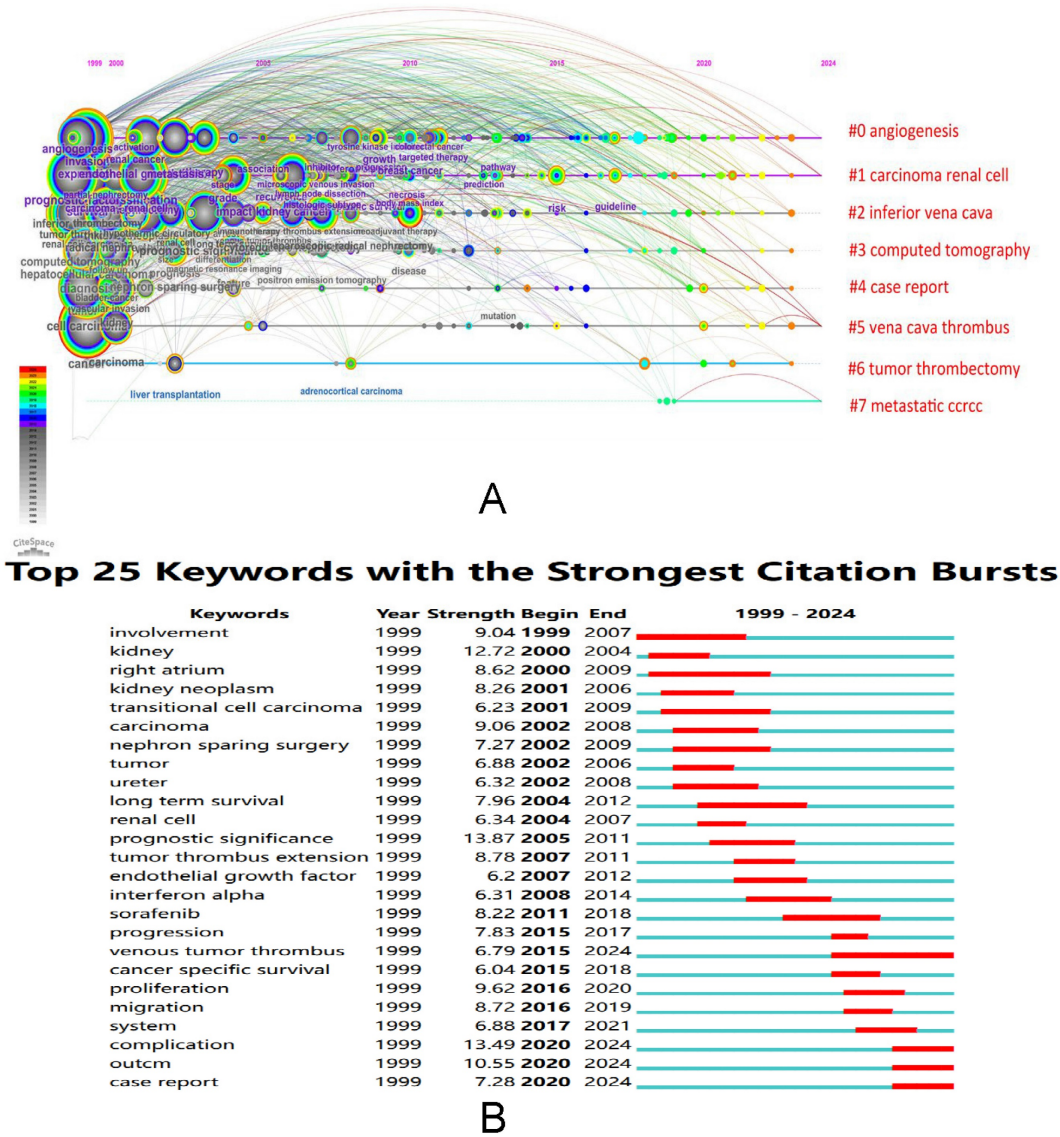
(A)The timeline view of the keywords in articles related to RCC with VTT, (B) the analysis of burst keywords in articles related to RCC with VTT.

The Timeline Viewer illustrates the dynamic evolution of research hotspots represented by keywords, offering insights into the temporal trends of research fields through clusters and the progression of research on hotspot keywords. Documents within a cluster are aligned on a common horizontal line, with time advancing from left to right, signifying the chronological sequence. The number of documents within a cluster emphasizes the depth and importance of research achievements in that particular field. **Figure 10A** visually presented the key stages and developmental trajectories of RCC with VTT research over time. Each cluster was identified by a cluster ID number, denoted as #0, #1, #2, and so forth. The figure showed eight clusters, which were angiogenesis, carcinoma renal cell, inferior vena cava, computed tomography, case report, vena cava thrombus, tumor thrombectomy, and metastatic ccRCC. From the figure, we can see that the clusters involved in the first decade were vena cava thrombus, inferior vena cava, and computed tomography. The keywords involved in 2010-2022 were angiogenesis, tumor thrombectomy, and metastatic ccRCC.

### Analysis of burst keywords

Keyword bursts showed an inspiring increase in different research interests in RCC with VTT. **Figure 10B** reflected the top 25 keywords with the strongest citation bursts. In particular, the keywords that appeared to burst in 2000-2010 were “involvement” (burst strength: 9.04), “kidney” (burst strength: 12.72), “right atrium” (burst strength: 8.62), “kidney neoplasm” (burst strength: 8.26), “transitional cell carcinoma” (burst strength: 6.23), “nephron-sparing surgery” (burst strength: 7.27), and “long term survival” (burst strength: 7.96). "Sorafenib" (burst strength: 8.22), "venous tumor thrombus" (burst strength: 6.79), "proliferation" (burst strength: 9.62), "migration" (burst strength: 8.72), "complication" (burst strength: 13.49), and "cancer-specific survival" (burst strength: 6.04) were the prominent burst keywords that emerged in 2011 and have remained relevant up to the present day.

## Discussion

### General information

In this study, we analyzed RCC with VTT documents using CiteSpace to visualize research results and progress. We conducted a quantitative analysis of basic information such as annual publication quantity, country, author, institution, and journal. In 1999, there were 33 documents published in this field, and this number increased to 143 by 2023, indicating an overall upward trend. The impact and quality of a paper in the field are often associated with the number of citations it receives. **Figure 2A** depicted the trend of cited references over time in this field. By statistically analyzing publications from various countries/regions and institutions, we can identify key contributors to RCC with VTT research and their collaborative relationships. The primary countries engaged in RCC with VTT research were the USA, China, Japan, Germany, and Italy. Among the top 10 institutions, 7 were located in the USA, while the remaining three were in China, notably the University of Miami, which had the highest publication count and h-index. The collaboration observed among countries and institutions were valuable for overcoming academic barriers and advancing research.

Of the top 10 authors, Ciancio G (72 papers) from the University of Miami Miller School of Medicine was the most published author, followed by Ma L (61 papers) and Liu Z (43 papers). This indicated that these three authors have made significant contributions in the field of RCC with VTT. In 2003, Professor Ciancio G reviewed the surgical techniques for treating RCC with VTT. He noted that VTT in RCC no longer adversely impacts survival and stressed that surgery was the sole curative treatment for this type of cancer. He emphasized that a proactive surgical approach is necessary when the VTT happens. The surgical strategy should be determined by the level of the VTT and underscored the importance of inducing cardiac arrest with cardiopulmonary bypass (CPB) during VTT resection 29. In 2021, he reported another retrospective study involving patients who underwent RN, tumor thrombectomy, and visceral resection for RCC 30. The five-year overall survival (OS) rate was 89.9% for patients with pancreaticoduodenal involvement and 75% for those with liver invasion. This study reaffirmed the importance of surgery in RCC with VTT and demonstrated the technical feasibility of the procedure with its acceptable complication rates, no mortality, and the potential for durable response. In a bi-institutional retrospective review, Ciancio G explored the impact of not reconstructing the IVC on renal function after resection of VTT, revealing that reconstruction of the IVC after resection of retroperitoneal tumors with VTT may not be necessary if sufficient collateral circulation is present 31. Ma L and Liu Z, from the Third Hospital of Peking University, conducted research on investigating ccRCC with VTT at the genomic, protein, and cellular levels. Their study on the genomic landscape of ccRCC revealed a prevalent mutational signature associated with aristolochic acid (AA) exposure in the VTT track. Mutations in BAP1 and SETD2 were also notably enriched 32, and proteins related to adhesion, migration, and invasion, such as L1CAM, were identified in the VTT track 33.

Subsequently, they elucidated the multicellular ecosystem of VTT in ccRCC using single-cell RNA sequencing 10. The presence of tissue-resident CD8+ T cells with a progenitor-exhaustive phenotype in the tumor thrombus was observed compared to the matched primary tumors. Additionally, macrophages, malignant cells, endothelial cells, and myofibroblasts from the tumor thrombus exhibited enhanced in extracellular matrix remodeling.

The most co-cited journals in the field of RCC with VTT were J UROLOGY (1330 citations), EUR UROL (1016 citations), and UROLOGY (938 citations). Out of the top 20 co-cited journals, 14 are situated in the Q1 JCR region. Notably, CA-CANCER J CLIN, with an IF of 254.7 and Q1 status, boasts the highest IF among these journals, while 8 journals have an IF exceeding 10. Journal citation reports profiles serve as journal-specific indicators concerning impact factors, rankings, and quartiles across various categories 34. Analyzing the distribution of literature sources can aid in identifying core journals within the relevant literature, assisting scholars in advancing scientific accomplishments. Most of the top 10 references centered on IVC VTT levels, survival time, a variety of surgical techniques, and related perioperative complications. Over the past two decades, substantial progress has been achieved in refining surgical methods and introducing innovative techniques. The high burst signal of references indicates a strong level of interest and time interval 35. Among the 20 references with the most significant citation bursts, the most frequently cited ones in recent years were primarily related to ICIs, TKIs, robot-assisted thrombectomy, and machine learning for predicting VTT based on imaging.

### Hotspots and frontiers

Analysis of high-frequency keywords serves as a valuable tool for identifying the prevailing trends within a specific research domain. In our study, we utilized the key co-occurrence analysis to pinpoint the primary directions and focal points in RCC with VTT, thereby elucidating the evolution and transitions in its thematic structure 36. Through cluster analysis based on keywords, we derived a cluster comprising major eight distinct colors. Subsequently, by delving into the top 25 keywords exhibiting the strongest citation bursts, we delineated the research hotspots and frontiers, highlighting the following key findings.

When dealing with RCC accompanied by vascular involvement, surgery remains the preferred treatment modality. In the case of RCC patients with VTT, surgical management demands rigorous preoperative planning. A crucial part of this preoperative evaluation is accurately pinpointing the location of the tumor thrombus 37. According to the Neves-Zinke classification, thrombi are designated as IIIa, IIIb, IIIc, and IIId, depending on their position: below the major hepatic veins, reaching the ostia of these veins, between the major hepatic veins and the diaphragm, or extending to the intrapericardial IVC while remaining infra-atrial 38, 39. For patients with level I thrombi, a traditional RN can be pursued. The open approach for RN with tumor thrombectomy often involves a midline, chevron, or subcostal incision. In scenarios involving level II thrombi, it is imperative to ensure thorough exposure and control of the infrahepatic and retrohepatic IVC prior to thrombectomy. Meanwhile, level III thrombi necessitate preoperative imaging for a comprehensive tumor-level characterization. Although the successful utilization of cardiopulmonary bypass (CPB) with circulatory arrest has been documented for the resection of high-level tumor thrombi 40, it carries risks such as coagulopathy, platelet dysfunction, and central nervous system complications 41. To circumvent the need for CPB, techniques like hepatic mobilization maneuvers have been employed to achieve complete intraabdominal resection of high-level thrombi 31. In recent years, laparoscopy and robot-assisted surgery have gained significant traction due to their minimally invasive nature and reduced incidence of postoperative complications. However, given the high-risk nature of operations involving level III-IV thrombosis, further exploration into the safety of these laparoscopic and robot-assisted surgical approaches is warranted 18.

While RN with tumor thrombectomy remains the gold standard treatment for advanced RCC, the utilization of immunotherapy agents for RCC with VTT has been a topic of growing interest in the literature. TKIs are the preferred targeted therapy that inhibits tumor growth and spread by targeting the von Hippel-Lindau (VHL) gene through vascular endothelial growth factor receptors. These receptors include VEGF receptor kinases, as well as PDGF receptor beta, c-KIT, and FLT3 42, 43. The utilization of neoadjuvant therapy to achieve complete or nearly complete resection in cases of locally advanced RCC is currently limited to sunitinib, sorafenib, and axitinib. Field constructed a clinical trial with a total of 53 patients who were administered sunitinib 50 mg/day for a 6-week circle. Initial analysis showed median primary tumor decreased in size from 8.1 to 6.8 cm, and the IVC tumor decreased by 1.3 cm. IVC tumor level decreased in 42.1% of enrolled patients 44. Karam included data on a total of 24 patients with biopsy-proven conventional T3a RCC 45, the disease volume was reduced by 28.3%, achieving partial responses in 11 patients (46%) after receiving axitinib neoadjuvant treatments for 12 weeks. In Hatiboglu's study 46, patients with locally advanced RCC were randomly assigned in a 3:1 ratio to receive either sorafenib (400 mg twice daily for 4 weeks) or a placebo. The sorafenib treatment group showed a statistically significant reduction (*P* < 0.05) in tumor size in 29% of cases, enabling the intervention to be carried out in a total of 75% of patients.

While TKIs have been the mainstay of treatment, the emergence of resistance has driven the development of new therapeutic strategies. ICIs have progressively reshaped the treatment paradigm in solid tumors, targeting molecules such as programmed cell death protein 1 (PD-1) and its ligand (PD-L1) 47. Combination therapy involving TKIs and ICIs has shown initial promising results, although the final overall survival in IMmotion151 showed that ICIs and TKIs tracks had similar results 48, the further outcomes of this alternative treatment need deeper investigated and are now gaining increasing recognition and interest. Many studies investigating the combination of TKIs with ICIs are currently underway, like Keynote-564 49, and Checkmate-914 50. These studies also involve supplementary correlative investigations aimed at elucidating the impact of the tumor microenvironment and identifying potential biomarkers that could help predict the tumor response to treatment 51.

Microvascular invasion (MVI), defined as the infiltration of cancer cells into blood vessel walls or the presence of cancerous emboli within vessel lumens, as one kind of vascular invasion at the pathological level, has gradually gained attention in recent studies (**Fig. 9**). Bengió RG 52 concluded that the presence of MVI demonstrated a strong unfavorable correlation with clinical symptoms, tumor size, nuclear grade, distant metastasis, and cancer-related mortality (*P*<0.05). Kroeger 53 also observed that MVI is one of the most critical factors associated with metastatic disease recurrence in patients with clinically localized disease. Due to the limited tissue volume, research on the sequencing of MVI and the identification of its corresponding biomarkers has not been extensively explored. Presently, the primary focus lies in investigating the correlation between MVI occurrence and various pathological traits like tumor volume, lymph node invasion, and renal fat invasion 54, 55. The findings indicate that as a potentially malignant pathological feature, the presence of MVI was observed to be associated with a high probability of poor prognosis 56.

There are several limitations in this study. Firstly, to ensure a high-quality bibliometric analysis, this study only relies on articles from the WoSC database, a renowned source for scientific publications across various research domains. Secondly, the data analysis is automated using software, which introduces the potential for unnoticed biases from machine algorithms. Thirdly, bibliometrics is unable to evaluate the individual study quality as citation metrics are time-sensitive, leading to newer articles potentially being less cited than older ones due to publication dates.

## Conclusion

Through detailed bibliometric analysis in RCC with VTT, this study assesses literature across various years, countries, institutions, authors, and journals to analyze theme development and future research trends. Our findings indicate that this special field started gaining attention in 2004. Current research focal points in this area predominantly encompass thrombectomy, tyrosine kinase inhibitors, immune checkpoint inhibitors, and microvascular invasion. Currently, thrombectomy complications, thrombectomy survival outcomes, and preoperative neoadjuvant immunotherapy are the frontiers of research in this field and are currently emerging.

## Supplementary Material

Supplementary table.

## Figures and Tables

**Table 1:** The top 10 countries and institutions contributed to RCC with VTT.

Rank	Country	Centrality	Count	Institutes	Centrality	Count
1	USA	1.06	676	Univ Miami (USA)	0.02	81
2	China	0.29	315	Mayo Clin (USA)	0.02	49
3	Japan	0.26	248	Peking Univ (CHN)	0.02	46
4	Germany	0.15	136	Univ Texas MD Anderson Canc Ctr (USA)	0.02	41
5	Italy	0.1	120	Peking Univ Third Hosp (CHN)	0	35
6	France	0.13	100	Chinese Peoples Liberat Army Gen Hosp (CHN)	0.03	31
7	Spain	0.08	89	Univ Calif Los Angeles (USA)	0.03	30
8	Canada	0.07	80	Mem Sloan Kettering Canc Ctr (USA)	0.03	25
9	UK	0.15	46	Cleveland Clin (USA)	0.04	25
10	Turkey	0.1	41	Emory Univ (USA)	0.08	25

**Table 2:** The top 10 authors and cited authors contributed to RCC with VTT.

Rank	Author	Centrality	Count	Cited author	Centrality	Count
1	Ciancio G	0.01	72	Blute ML	0.39	360
2	Ma L	0	61	Ciancio G	0.52	307
3	Liu Z	0	43	Neves RJ	0.29	263
4	Zhang S	0	40	Motzer RJ	0.13	257
5	Gonzalez J	0	35	Skinner DG	0.16	229
6	Ma X	0	31	Ljungber GB	0.41	212
7	Liu C	0	30	Klatte T	0.04	181
8	Zhang H	0	30	Zisman A	0.27	157
9	Wang G	0	29	Jemal A	0.05	152
10	Zhang X	0	28	Rini BI	0.03	133

**Table 3:** The top 10 highly cited articles and top 10 keywords of RCC with VTT

Rank	Articles	Centrality	Citations	Keyword	Centrality	Count
1	Moinzadeh A 14, 2004	0.01	52	renal cell carcinoma	0.52	896
2	Wagner B 15, 2009	0.06	49	cancer	0.11	473
3	Kim HL 16, 2004	0.01	48	tumor thrombus	0.07	349
4	Ciancio G 17, 2007	0.02	43	inferior vena cava	0.02	316
5	Chopra S 18, 2017	0.01	42	radical nephrectomy	0.08	288
6	Blute ML 19, 2004	0.03	42	survival	0.27	284
7	Martínez-Salamanca JI 20, 2011	0.02	41	surgical management	0.41	266
8	Wang BJ 21, 2016	0.01	40	nephrectomy	0.03	240
9	Méjean A 22, 2018	0.02	37	extension	0.62	240
10	Ljungberg B 23, 2015	0	37	prognosis	0.28	214
