# Supplementary material for: Bibliometric analysis of renal cell carcinoma with venous tumor thrombus
Source: Int J Med Sci. 2024 Aug 12;21(11):2094–108. doi: 10.7150/ijms.98359 (PMC11373542; doi:10.7150/ijms.98359)
Supplement: Supplementary file 1 — Supplementary table. [file ijmsv21p2094s1.pdf]

Supplementary Table 1 The top 10 co-cited journals about RCC with VTT

Rank	Cited journal	Citations	IF	Centrality	Q	Country
1	J UROLOGY	1330	5.6	0.45	Q1	USA
2	EUR UROL	1016	20	0.21	Q1	Netherlands
3	UROLOGY	938	2	0.2	Q3	USA
4	BJU INT	868	4	0	Q1	UK
5	NEW ENGL J MED	498	176.08	0.08	Q1	USA
6	J CLIN ONCOL	489	45.4	0	Q1	USA
7	CANCER-AM CANCER SOC	467	6.07	0.04	Q1	USA
8	UROL ONCOL-SEMIN ORI	425	2.882	0.22	Q2	Netherlands
9	ANN SURG	412	10.1	0.38	Q1	USA
10	BRATISH J UROL	386	--	0.33	--	UK

IF, impact factors; Q, first quartile of the journal citation reports
